# The Efficiency of the Kannada Digit Triplet Test to Identify Older Adults with Mild-to-Moderate Sensorineural Hearing Loss

**DOI:** 10.1055/s-0046-1818550

**Published:** 2026-04-24

**Authors:** Salman Safeer, Mohan Kumar Kalaiah

**Affiliations:** 1 Department of Audiology and Speech Language Pathology, Kasturba Medical College Mangalore, Manipal Academy of Higher Education, Manipal, India

**Keywords:** Kannada digit triplet test, hearing loss, sensorineural, speech in noise, sensitivity, specificity

## Abstract

**Introduction:**

Hearing loss, especially age-related, affects speech perception and quality of life. Despite its prevalence, many individuals remain undiagnosed due to low awareness and limited access to screening tools. The Digit Triplet Test (DTT) is a proven speech-in-noise screening tool adapted into many languages. In India, the Kannada DTT was developed to address the need for a regional screening method. The present study aims to evaluate the effectiveness of the Kannada DTT to identify older adults with hearing loss.

**Objective:**

To determine the sensitivity and specificity of the Kannada DTT in identifying older adults with mild-to-moderate sensorineural hearing loss (SNHL).

**Methods:**

A total of 125 native Kannada-speaking participants aged 50 to 84 years were divided into 2 groups: 57 with normal hearing and 68 with mild-to-moderate hearing loss. Pure tone audiometry and speech identification scores were recorded. Participants underwent the Kannada DTT monaurally in both ears using an adaptive protocol.

**Results:**

The DTT scores were significantly poorer in the hearing loss group. A moderate positive correlation (r ≈ 0.68) was observed between pure tone average and DTT score. The receiver operating characteristic (ROC) curve analysis revealed that a DTT cutoff score of −11.05 dB provided the best classification accuracy, with sensitivity and specificity of 79.4% and 82.5% for the right ear, and 83.8% and 86% for the left ear, respectively.

**Conclusion:**

The Kannada DTT demonstrates good sensitivity and specificity in identifying older adults with mild-to-moderate SNHL and can be considered a reliable screening tool for early detection.

## Introduction

Hearing impairment significantly impacts speech perception abilities. 1 Additionally, some individuals with normal hearing struggle to understand speech in challenging auditory environments. Studies have explored the prevalence of hearing loss across different age groups and genders. Research on elderly populations has shown that mild hearing impairment affects 35.7% of individuals, moderate impairment (35–49 dB) is observed in 8.5%, moderately severe impairment (50–64 dB) in 1.8%, and severe impairment (65–70 dB) in 0.1% of cases. 2 In India, community-based research estimated hearing loss prevalence to range between 6 and 26.9%, with disabling hearing loss affecting 4.5 to 18.3% of the population. 3

Age-related sensorineural hearing loss is the most common type, typically progressive and bilateral, initially affecting high frequencies and often going unnoticed by the individuals affected. 4 Despite its impact on communication and quality of life, help seeking remains low due to denial, social stigma, and lack of awareness. 5 Integrating hearing screening into primary care could facilitate early detection and intervention.

Speech-in-noise (SIN) tests are valuable tools for evaluating speech perception under adverse listening conditions. 6 Various SIN methods have been developed using different stimuli and scoring strategies. 7 8 Digit-based tests are particularly effective due to their simplicity, language independence, and strong correlation with pure-tone thresholds. 9 10 11 The Digits-in-Noise (DIN) test offers efficient and user-friendly screening for hearing loss. 12 13

The Digit Triplet Test (DTT), introduced by Smits et al. in 2004, was originally developed as an automated self-test using 3-digit sequences presented in noise, referred to as the Dutch National Hearing Test. 14 This test estimates the signal-to-noise ratio (SNR) at which 50% of digit triplets are correctly identified. The DTT has proven to be effective in identifying speech comprehension difficulties in noisy settings, a common complaint among those with hearing loss. 15 It has demonstrated high sensitivity and specificity (∼ 80%) in detecting hearing impairment. 16

The success of DTT has led to its adaptation into multiple languages, facilitated by initiatives such as the European HearCom project. The International Collegium of Rehabilitative Audiology (ICRA) established guidelines in 2015 for the development of speech-based tests like the DTT in various languages. 17 The test has since been translated into English, Polish, French, German, American English, Flemish, Finnish, Australian English, Turkish, South African English, and Chinese. Studies on DTT adaptations across languages confirm its efficiency and validity for assessing speech intelligibility in noise and determining speech reception thresholds. 13 The DTT has evolved over time, expanding across different screening platforms (telephone, internet, tablets, smartphones) to reach diverse populations, including children. Recent advancements include diagnostic adaptations for cochlear implant users, with ongoing research aimed at enhancing sensitivity, reliability, and practical application. 16

In India, researchers developed the Kannada DTT to address the lack of such a tool in the Kannada language. Initially, bisyllabic digits were recorded and adjusted to match speech reception thresholds (SRTs). Five lists of 27 triplets each were generated and evaluated for homogeneity using SRT measurements in speech-shaped noise. Statistical analysis confirmed that all lists provided equivalent difficulty levels, establishing the Kannada DTT as a reliable screening tool for assessing auditory function. 18 The selection of digits for the test was based on their widespread familiarity, even among young children, thus minimizing language and age-related barriers. 11 There is a need to validate the Kannada DTT for using as a hearing screening tool to identify individuals with hearing loss. Thus, the current study aims to evaluate the effectiveness of the Kannada DTT to identify older adults with hearing loss. The objective of the present study is to determine the sensitivity and specificity of the Kannada DTT in identifying adults with mild-to-moderate SNHL.

## Methods

**Fig. 1:**
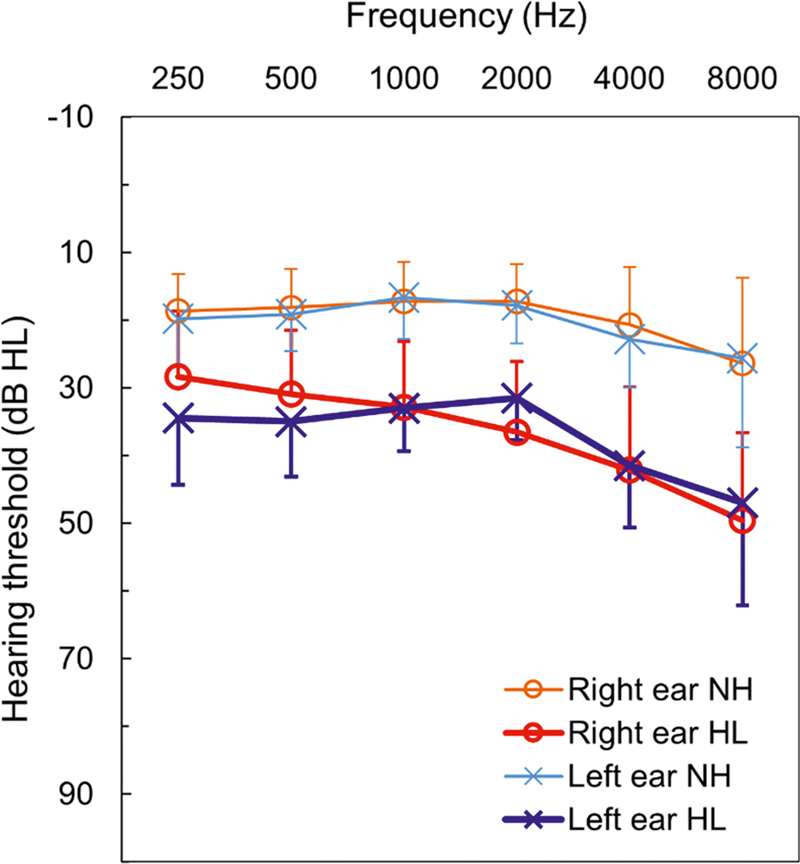
Mean hearing threshold and standard deviation (error bars) across frequencies for normal hearing (NH) and hearing loss (HL) groups.

A total of 125 older adults (70 males and 55 females), aged between 50 and 84 (mean: 59.8 ± 8.09) years, participated in the study. Based on their hearing sensitivity, the participants were categorized into the normal hearing (NH) or the hearing loss (HL) group. The NH group consisted of 57 individuals (27 males, 30 females) aged between 50 and 68 (mean: 55.8 ± 4.55) years, all of whom had pure-tone thresholds ≤ 25 dB HL at octave frequencies ranging from 250 Hz to 8,000 Hz. These participants reported no history of otological symptoms such as ear pain, ear discharge, or related complaints. They also showed Speech Identification Scores (SISs) of 80% or higher in quiet for both ears and were native Kannada speakers with the ability to read, speak, and understand the language fluently. Additionally, they reported no difficulty in understanding speech in quiet or noisy environments. Participants were excluded from this group if they had any history of otological or neurological dysfunction, exhibited signs of auditory processing or cognitive deficits, or reported communication difficulties in challenging listening conditions. The HL group included 68 individuals (43 males, 25 females) aged between 50 and 84 (mean: 63.29 ± 8.83) years, all presenting with bilateral mild-to-moderate hearing loss, with a pure-tone average (PTA) between 26 and 55 dB HL ( Fig. 1 ). Within this group, 53 participants had SNHL, 10 had conductive hearing loss (CHL), and 5 had mixed hearing loss (MHL). All participants in this group were also native Kannada speakers with the ability to read, speak, and comprehend the language. Exclusion criteria for this group included the presence of auditory processing, neural, or cognitive deficits and self-reported communication challenges in adverse listening environments. Patient consent was obtained before each data collection. The present study was conducted after obtaining approval from the institutional ethics committee of Kasturba Medical College, Mangalore (protocol number: IEC KMC MLR 04/2024/205).

### Procedure

Participants with no complaint of hearing loss initially underwent a basic audiological evaluation, which included pure-tone and speech audiometry. Prior to the evaluation, a detailed case history was collected. The hearing threshold was estimated using the modified Hughson and Westlake procedure on a calibrated GSI 61 (Grason-Stadler). This clinical audiometer, equipped with standard accessories, was utilized to estimate the air conduction and bone conduction hearing thresholds of participants. The speech identification score in quiet was measured by presenting speech at 40 dB SL (reference: PTA) using the Kannada Word Recognition Test. 19 Finally, the Kannada DTT was conducted.

### Kannada Digit Triplet Test

The Kannada DTT was administered using a Lenovo laptop (Lenovo Group Limited) with Intel Core i3 64 bit processor (Intel Corporation), Windows 10 operating system (Microsoft Corp.) and MATLAB software (Matrix Laboratory) along with Sennheiser HD 280 PRO headphone (Sennheiser electronic GmbH & Co. KG). Prior to administration of the DTT, participants were instructed that, in each stimulus block, they would hear three digits presented along with background noise, and they were to listen carefully and respond by pressing the digits on the response pad in the same order as they were presented. They were also informed that they could guess the digits if they were unable to recognize one or more digits in the block. Practice trials were given to only those patients who had confusion and doubt about how to respond.

The digits were presented monaurally along with noise to each participant at the most comfortable level of the participant. Initially, digit triplets were presented at poorer SNR (that is, −20 dB), in case of a wrong response, the same triplet was presented again after increasing the SNR by 2 dB until a correct response was obtained. After obtaining the first correct response, the noise level was varied adaptively using a simple up-down method with a step size of 2 dB. 20 The remaining triplets were presented using a simple up-down procedure until the end of the list. The response was considered correct when the participant identified all digits in the triplet in the same order as presented. The DTT score (SNR-50) was calculated as the arithmetic mean of the mid-points of all reversals.

### Statistical Analysis

The pure-tone hearing threshold for each participant across different frequencies, along with speech identification and DTT scores, was recorded in a Microsoft Excel sheet (Microsoft Corp.). Statistical analysis was conducted using Jeffrey's Amazing Statistics Program (JASP; free and open-source), version 0.19.3 and the IBM SPSS Statistics for Windows (IBM Corp.) version 29.0. Initially, descriptive analysis was performed on the data. The Shapiro-Wilk test of normality was applied to determine whether the PTA and DTT scores for both ears in both groups followed a normal distribution. The Mann-Whitney U test was used to examine the impact of group differences on DTT scores. Additionally, Spearman's correlation analysis was conducted to assess the relationship between PTA and DTT scores. Receiver operating characteristics (ROC) curve analysis was performed to establish the optimal cutoff value for the Kannada DTT in distinguishing individuals with normal hearing from those with hearing loss. Sensitivity, specificity, and the area under the curve were also calculated to evaluate the overall effectiveness of the test.

## Results

### Pure Tone Audiometry

**Fig. 2:**
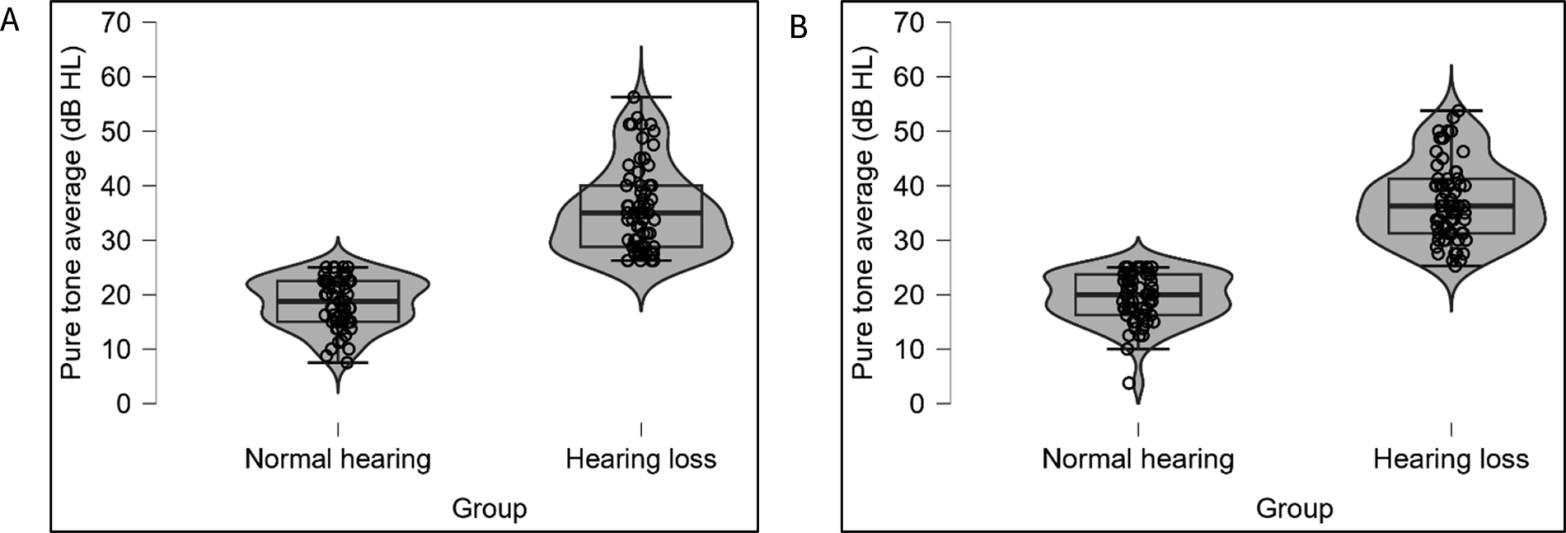
Violin plots of pure tone average for normal hearing and hearing loss groups in the right ear (panel A) and left ear (panel B).

A total of 68 individuals with hearing loss participated in the study. Among them, 53 participants had SNHL, 10 participants had CHL, and the remaining 5 participants had MHL. Additionally, 53 participants had mild hearing loss and 15 had moderate hearing loss in the right ear, and in the left ear, 50 participants had mild hearing loss and 18 had moderate hearing loss. Table 1 shows the mean PTA in the right and left ears for both groups. Fig. 2 shows the violin plots of PTA for normal hearing and hearing loss groups in both ears. It displays the full distribution of the data, including the 25th and 75th percentiles and the median. Individual data points are also plotted for each group and both ears. The PTA for participants in the normal hearing group was between 7.5 and 25 dB in the right ear and between 3.75 and 25 dB in the left ear. Similarly, the PTA for participants in the hearing loss group was between 26.25 and 56.75 dB in the right ear and 25.25 and 53.75 dB in the left ear. Table 1 also shows the mean PTA of participants with CHL, SNHL, and MHL in both ears.

**Table 1:** Mean pure tone average and standard deviation (in parenthesis) for the right ear, left ear, and both ears across normal hearing and hearing loss groups

Group	Normal hearing	Hearing loss			
		CHL	SNHL	MHL	Total
**Right ear**	18.4 (4.6)	33.8 (4.4)	36.3 (8.5)	33.0 (7.4)	35.7 (8.0)
**Left ear**	19.3 (4.6)	35.0 (4.8)	37.8 (7.4)	35.5 (8.8)	37.2 (7.2)
**Both ears**	18.9 (4.6)	34.4 (4.6)	37.0 (8.0)	34.3 (7.8)	36.4 (7.6)

**Abbreviations** : CHL, conductive hearing loss; DTT, Digit Triplet Test; MHL, mixed hearing loss; SNHL, sensorineural hearing loss.

**Note** : Pure tone average in dB HL.

### Digit Triplet Test

**Fig. 3:**
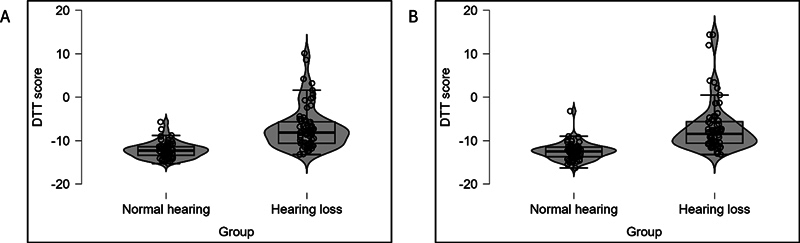
Violin plots of Digit Triplet Test (DTT) scores for normal hearing and hearing loss groups in the right ear (panel A) and left ear (panel B).

Table 2 shows the mean DTT scores for both ears of participants in both groups. Figs. 3 4 show the violin plots of DTT scores in the right and left ear for both groups. The mean DTT score of both ears was similar in both groups. Further, the DTT score was poorer in the hearing loss group compared with the normal hearing group. The lowest DTT scores in the normal hearing group were −15.3 dB in the right ear and −16.3 dB in the left ear, and the highest scores were −5.7dB in the right ear and −3.2dB in the left ear. Similarly, the lowest DTT scores in the hearing loss group were −13.2 dB in the right ear and −13.2 dB in the left ear, and the highest scores were 10.1 dB in the right ear and 14.4 dB in the left ear.

**Table 2:** Mean Digit Triplet Test score and standard deviation of both ears in the normal hearing and hearing loss groups

Group		Normal hearing	Hearing loss			
			CHL	SNHL	MHL	Total
**Right ear**	Mean	−12.21	−7.3	−7.0	−8.0	−7.11
	SD	1.85	5.3	5.3	2.6	4.88
**Left ear**	Mean	−12.46	−7.9	−6.8	−7.9	−7.05
	SD	2.01	6.2	5.9	4.0	5.8
**Both ears**	Mean	−12.3	−7.6	−6.9	−7.95	−7.08
	SD	1.92	4.83	5.6	3.19	5.34

**Abbreviations:** CHL, conductive hearing loss; MHL, mixed hearing loss; SNHL, sensorineural hearing loss; SD, standard deviation.

**Note** : Digit Triplet Test score in dB.

**Fig. 4:**
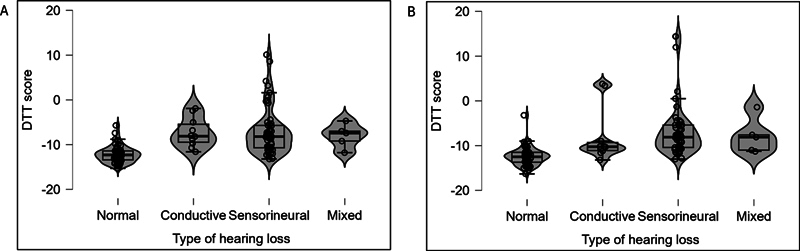
Violin plots of DTT scores in the right ear (panel A) and left ear (panel B) for normal hearing group and different types of hearing loss in hearing loss groups.

Paired *t* -test was conducted to determine if there was a significant difference in the mean DTT scores between the right and left ears in the normal hearing group. The results indicated no significant difference between the ears (t[56] = 1.141; *p* = 0.259). Similarly, the Wilcoxon signed-rank test was performed to examine whether the mean DTT scores differed between ears in the hearing loss group. The findings showed no significant difference between the ears (t[67] = 1182; *p* = 0.791). The Mann-Whitney test was then used to assess whether the mean DTT scores of the right and left ears significantly differed between groups. The results revealed a significant difference in mean DTT scores between groups for both ears (right ear [W = 436.5; *p* < 0.001]; left ear [W = 357.5; *p* < 0.001]).

### Relationship between the DTT score and PTA

**Fig. 5:**
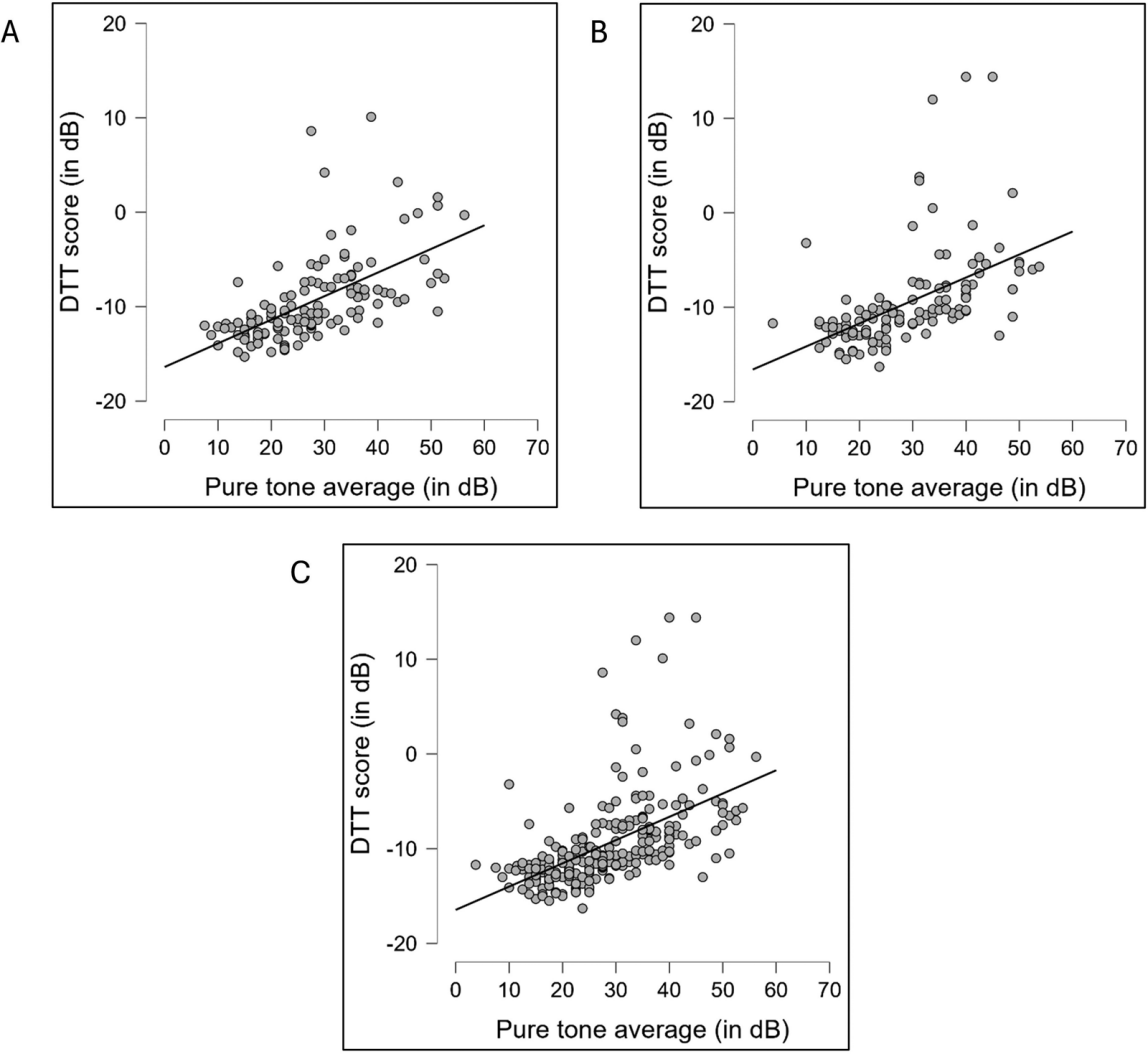
Scatter plots show the relation between the DTT score and pure tone average (PTA) of the right ear (panel A), the DTT score and PTA of the left ear (panel B), and the DTT score and PTA of both ears together (panel C).

A Spearman's correlation analysis was performed to investigate the relationship between PTA and DTT scores in both ears. Results showed a significant moderate positive correlation between the DTT score and PTA in the right ear, the DTT score and PTA in the left ear, and the DTT score and PTA of both ears. Table 3 shows the results of the Spearman's correlation analysis. Fig. 5 shows scatter plots representing the relationship between the DTT score and PTA for both ears.

**Table 3:** Spearman's rho, *p* -value, and confidence intervals of the Spearman's correlation analysis between the DTT score and pure tone average

				
	Spearman's rho	*p*	95%CI: lower bound	95%CI: upper bound
**PTA and DTT score of the right ear**	0.699	< 0.001	0.597	0.779
**PTA and DTT score of the left ear**	0.681	< 0.001	0.575	0.765
**PTA and DTT score of both ears together**	0.688	< 0.001	0.617	0.748

**Abbreviations** : DTT, Digit Triplet Test; PTA, pure tone average.

### Sensitivity and Specificity of the Kannada Digit Triplet Test

**Fig. 6:**
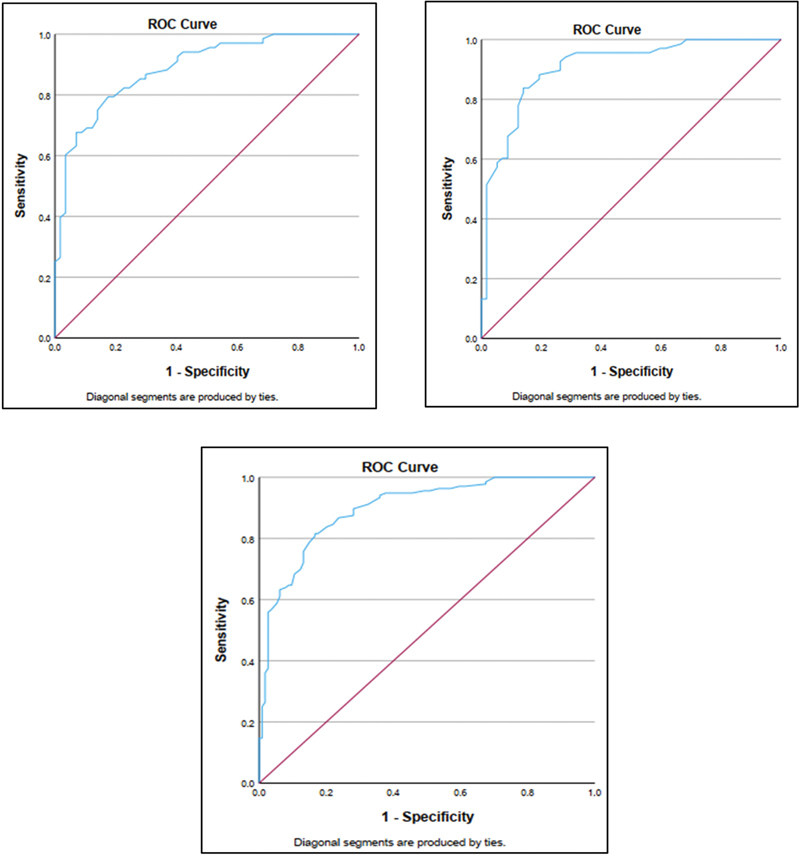
Receiver operating characteristic curve showing the sensitivity and specificity of the Kannada DTT score for different cutoff values in the right and left ears and for both ears together.

The sensitivity and specificity of the Kannada DTT depend on the cutoff value of the DTT score used to classify individuals as having normal hearing or hearing loss. The sensitivity and specificity were computed for three cutoff values. The mean DTT score minus two standard deviations (2SD), the 10th percentile, and the optimal threshold determined by the ROC curve analysis. The ROC curve was used to identify the optimal cutoff value for classifying individuals into the normal or hearing loss group. The ROC curve obtained is shown in Fig. 6 . The ROC curve data includes several cutoff values along with its corresponding sensitivity and specificity values. The ROC curve analysis showed that the area under the curve (AUC) was 0.887 for the right ear, 0.908 for left ear, and 0.898 for both ears. The sensitivity, specificity, and overall accuracy for three cutoff values of the DTT score are shown in Table 4 . The cutoff value −11.05 based on the ROC curve analysis had highest accuracy for classification of participants into normal or hearing loss. Thus, it could be considered as a good compromise between high sensitivity and high specificity for the Kannada DTT.

**Table 4:** Digit Triple Test score cutoff values and corresponding sensitivity and specificity rates of the Kannada DTT for detecting individuals with hearing loss > 25 dB HL averaged over 0.5, 1, 2, and 4 kHz

		DTT score cutoff value (dB)	Sensitivity (%)	Specificity (%)	Accuracy
Right ear	Mean + 2SDs	−11.75	88.2	63.2	75.7
	Highest accuracy in ROC analysis	−11.05	79.4	80.7	80.5
	10th percentile	−10.05	69.1	89.5	79.3
Left ear	Mean + 2SDs	−11.90	95.6	68.4	92.5
	Highest accuracy in ROC curve analysis	−11.05	83.8	86	84.9
	10th percentile	−10.35	70.6	87.7	79.15
Both ears	Mean + 2SDs	−11.85	93.4	64	78.7
	Highest accuracy in ROC curve analysis	−11.05	81.6	83.3	82.45
	10th percentile	−11.65	89.7	71.9	80.8

**Abbreviations** : DTT, Digit Triplet Test; SD, standard deviation; ROC, receiver operating characteristic.

## Discussion

The results of the present study demonstrated that participants in the hearing loss group had poorer DTT scores compared with those with normal hearing. Similar outcomes have been documented in various studies. 2 15 21 22 23 It is well established that hearing loss impacts speech perception in noisy environments. 1 Research also indicates that individuals with hearing impairment require a higher SNR to achieve performance levels comparable to those with normal hearing sensitivity. The decline in performance among individuals with hearing loss is primarily attributed to reduced audibility and suprathreshold auditory processing deficits caused by the impairment. 24

Correlation analysis showed a significant, moderately positive correlation (r = 0.68) between the DDT score and PTA. In consonance with the findings of the present study investigations have reported a moderate correlation between the PTA and DTT score. 25 26 27 Vlaming, in 2014, reported a correlation coefficient of 0.64 among individuals with high frequency hearing loss. 26 Similarly, Denys, in 2016, reported a correlation coefficient of 0.65 and 0.64 for the right and left ears respectively among children who did not pass in the DTT. 25 However, across investigations, the correlation coefficient was found to be between 0.37 and 0.92. 2 12 23 26 28 29 Furthermore, the correlation coefficient for DTT across languages such as Dutch, French, American English, Australian English, Turkish, South African English and Dutch-Flemish was also similar. 14 29 30 31 32 33

The ROC curve analysis showed that the DTT score cutoff value of −11.05 dB resulted in the highest accuracy for classification of individuals into normal hearing or hearing loss. The sensitivity and specificity at this cutoff value were 79.4% and 82.5% for the right ear, 83.8% and 86% for the left ear, and 81.6% and 83.3% when both ears were taken together. In comparison to the findings of the present investigation, studies have reported sensitivity and specificity ranging from 75 to 95% and 67 to 98%, respectively, in older adults. 2 12 23 30 Similarly, the cutoff value for classifying individuals into normal hearing or hearing loss was between −8.5 dB to −16.2 dB. 2 12 15 21 22 23 26 34 The sensitivity and specificity of the Kannada DTT was comparable to the performance of the DTT across languages such as American English, South African English, and Dutch-Flemish languages. 2 12 23 26 27 30 33 35 Thus, the performance of the Kannada DTT was comparable to the performance of the DTT reported in the literature.

The findings of the present investigation show that the Kannada DTT is an effective tool for the identification of older adults with hearing loss. Studies have shown that DTT could be used for hearing screening over telephone and apps can be developed for smartphone use to screen hearing loss. Hence, it has potential for becoming the test of choice for carrying out large-scale hearing screening.

## Conclusion

The present investigation shows that the Kannada DTT can be considered an effective tool for the identification of older adults with hearing loss, thus suggesting that it is a promising tool for the screening of hearing loss in older adults. A larger sample size could have resulted in greater confidence or accuracy for the sensitivity and specificity of the Kannada DTT. In the future, the Kannada DTT could be administered to other populations, such as children and young adults, to establish the cutoff values for respective populations.
